# Documentary Assessment of the Abilities of Kyrgyzstan's Research Ethics Committees During Public Health Emergency and Non-Emergency Situations

**DOI:** 10.1177/15562646231176711

**Published:** 2023-05-18

**Authors:** Tamara Kudaibergenova, Muiz Ibrahim, Nityanand Jain, Janis Vetra

**Affiliations:** 1 Department of Public Health and Healthcare, 185188 I.K. Akhunbaev Kyrgyz State Medical Academy, Bishkek, Kyrgyzstan; 2 475774 Faculty of Medicine, International Higher School of Medicine, Bishkek, Kyrgyzstan; 3 Faculty of Medicine, 475768 Riga Stradinš University, Riga, Latvia

**Keywords:** research ethics committees, public health emergency, clinical trials, documentary analysis, legal framework, Kyrgyzstan

## Abstract

Institutional Research Ethics Committees (RECs) play crucial roles in the impartial and competent review of scientific research, particularly during public health emergencies. In this report, we examined their ability and capacity to provide this basic service during public health emergencies and non-emergency situations. Our qualitative documentary analysis revealed that there are currently no legal regulations guiding the activities of Kyrgyz RECs during public health emergencies. In addition, major policy gaps exist in how RECs should operate in non-emergency circumstances. This lack of guidance highlights the urgent need to develop and implement ethical guidelines to meet the evolving needs of such emergencies. Our findings underscore the growing urgency of supporting capacity building of RECs to respond effectively to future pandemics and other public health crises.

## Introduction

The recent surge in public health emergencies has highlighted the urgent need for being better prepared to address crucial research questions, especially in the context of a public health emergency. Kyrgyzstan, a country vulnerable to natural disasters such as earthquakes and landslides, is listed in the World Health Organization's (WHO) Health Emergencies Program and is considered a priority country in the European Region (WHO, 2019). Furthermore, current local socioeconomic conditions pose potential threats to the fragile health system. The country is endemic to various bacterial and viral infections including plague and anthrax and suffers from the increasing burden of multi-drug resistant tuberculosis and HIV. In addition, the country is vulnerable to transboundary infections due to the high influx of people from neighboring territories (Гаврилова et al., 2017). Given these circumstances, the national healthcare system must respond swiftly to such threats and develop resilience by undertaking research-related activities.

The knowledge and experience gained from well-designed and well-conducted research studies leading up to, during, and after an emergency, are critical for fostering and developing future capabilities and competencies to achieve the overarching goals of preparedness and response. An integrated approach to research in the context of emergencies is a prerequisite, including the development of the necessary infrastructure to strengthen the research response to emergencies (Lurie et al., 2013). Research ethics committees (RECs) must be central to such a response framework for future epidemic responses, especially for low- and middle-income countries (Al Tajir, 2018; Bain et al., 2018). Noticeably, research involving public health interventions, or research conducted during emergencies such as natural disasters and disease outbreaks, faces unique ethical challenges (Al Tajir, 2018).

Public health emergencies are accompanied by a hurried and panicked atmosphere in which researchers rush to obtain new REC authorizations for their research proposals. This increases the pressure on the RECs to expedite the review of proposals and can lead to superficial reviews (Bryzgalina, 2020; WHO, 2020a). In addition, research applications related to public health emergencies often need additional scrutiny in the following five domains – experience and awareness of researchers, interests and rights of participants, the societal value of the research, organization of review, and the distinction between research and non-research applications (Mezinska et al., 2016). Non-research proposals, including studies planned to improve quality and public health interventions with no initial intention to publish, are particularly difficult to manage and should never be self-evaluated by the investigators (Al Tajir, 2018).

A recent documentary review aimed at comparing national and international guidelines on disaster research ethics, found only 14 eligible open-access documents published between 2000 and 2014 (Mezinska et al., 2016). Amongst these documents, merely five were issued by national authorities (two each from Canada and USA and one from India). Although the study included only English language documents, these findings highlight the global scarcity of national regulations on research practices in emergencies (even amongst anglophone countries). A lack of national guidelines and awareness limits the capacity of the RECs in such situations, even more so in low- and middle-income countries which suffer from “ethics dumping” (Schroeder et al., 2018).

The present study, hence, aims to explore the capabilities of RECs in Kyrgyzstan to function in situations of rapidly developing public health emergencies. In our opinion, an institution requires an adequate legal framework to function properly and appropriately. Therefore, the aim of our study was to determine whether local RECs have such legal frameworks to provide an ethical review of research proposals during public health emergency and non-emergency situations.

## Methods

A qualitative documentary analysis of pre-existing textual data was performed (Bowen, 2009; Morgan, 2022). Relevant information from the official websites/online sources of the legal authorities involved in the regulation of biomedical research in Kyrgyzstan was extracted, collected, and archived. The available material was perused to select documents that fulfilled the following four criteria – authenticity, credibility, representativeness, and meaning (Flick, 2018). The authenticity of a document was confirmed by looking at whether it was a primary source or not, followed by confirmation of the date, location, and authorship of publication (Kridel, 2015). Credibility and representativeness were determined by the extent to which the source was free from error and distortion (Dunne et al., 2016).

For data collection, we employed purposive sampling. Only open-access (OA) documents were collected using the desk research technique that dealt with legal regulations of biomedical research. Next, we benchmarked and structured the collected documents using the definition of legal frameworks - “a set of documents that include the constitution, legislation, regulations, and contracts, where documents relate to one another and are referred to as a legal hierarchy that has a pyramid order: constitution, legislation, regulations, contracts” (NRGI, 2015).

The following themes were included for the selected documents: ethics, research ethics, research, biomedical research, clinical trials, behavioral research, psychological research, public health research, public health emergency research, research ethics committee, bioethics committee, research participant protection, consent, and/or Kyrgyzstan. For documentary analysis, reflexive thematic analysis was done and connecting themes were identified for drawing conclusions.

## Results

Our analysis revealed that currently Kyrgyz RECs solely regulate studies in the field of biomedicine. According to the Law of the Kyrgyz Republic (dated June 16, 2017, No. 103) “On science and on the foundations of the state scientific and technical policy” Chapter 3. “The system of science, scientific and scientific-technical organizations”, Article 9, the national scientific ecosystem includes five key pillars including:

1. The Government of the Kyrgyz Republic (https://www.gov.kg)
2. Authorized state body in the field of science (Ministry of Education and Science; (https://edu.gov.kg)
3. State and non-state scientific and scientific-technical organizations (National Academy of Sciences of the Kyrgyz Republic; https://naskr.kg/ky/, scientific departments of the higher educational institutions, sectoral and intersectoral research organizations, research centers and technology parks, research and production associations, experimental stations, scientific bases, stations, and laboratories)
4. National Attestation Commission of the Kyrgyz Republic (https://vak.kg)
5. Scientific workers.

Additionally, the Ministry of Justice (http://cbd.minjust.gov.kg), Ministry of Health and its Bioethics committee (https://med.kg), and the Department of Pharmaceutical Provision and Medical Equipment of the Ministry of Health (http://www.pharm.kg) play a crucial part in the national research ethics review system.

### Kyrgyzstan National Constitution

The constitution is the foundational document establishing the basic structure of government and prevails over all other legal instruments. Chapter VI of the constitution guarantees the rights and freedoms of all humans and citizens. Article 6 claims that medical, biological, and psychological interventions on people without their voluntary consent, expressed, and duly certified, are prohibited.

### Policies and Legislation

National policies and laws provide a coherent set of strategies and rules governing behavior in the research sector. Some policies are approved by the national legislature, while others are promulgated only by the executive branch. Legislation, on the other hand, is a legally binding set of rules that govern the vision set in a policy. In Kyrgyzstan, the procedure for conducting clinical and biomedical research and applying new methods of diagnosis and treatment is determined by the government, keeping in mind that the interests of individuals participating in clinical trials must always prevail over the interests of science and society (Table 1).

**Table 1.** Laws That Govern Scientific Research Ethics in Kyrgyzstan.

Law	Law Number; Year of Adoption	Description
Law on science and on the foundations of the state scientific and technical policy	103; 2017	Distinguishes the applied scientific research from fundamental scientific research without further clarification of the type of research. Article 17 claims that the scientific and scientific-technical workers are responsible for violation of professional ethics and moral rules.
Law on the protection of the health of citizens in the Kyrgyz Republic	06; 2005 (amended 2017)	Article 34 states that clinical and biomedical research are carried out on individuals with their written consent. The intervention is terminated at any stage at the request of the participants and in cases of a threat to their health. The law lacks definitions of clinical and biomedical research in the document. Article 72 states that in case of violation of the patient's rights, they may file a complaint directly with the head or other official of the healthcare organization in which they receive medical care, with the relevant professional medical public organizations or with the court. There are no guidelines protecting patients in cases where an intervention is suspended at the request of the participant or because of adverse reactions.
Law on Circulation of Medicines	165; 2017	Articles 24 and 25 of Chapter 7 regulate preclinical studies and clinical trials of new drugs. Article 24 states that preclinical trials should be conducted per the rules of good laboratory practice and the requirements for conducting trials prescribed by law in international treaties to which the Kyrgyz Republic is a party. Violations in conduction of trials entails liability. Trials should be terminated in the event of a threat to the life or health of participants along with provision of adequate compensation. Article 25 states that the participation of an individual in clinical trials is voluntary. Clinical trials are conducted with the written consent of an individual or their legal representative to participate in clinical trials. Article 4 mentions that an individual participating in clinical trials has the right to refuse to participate at any stage of the trial. Article 5 prohibits clinical trials involving minors, military personnel, persons serving sentences in places of deprivation of liberty, as well as on persons in custody in pre-trial detention centers, pregnant women, mentally ill and/or recognized as legally incompetent by the law.
Law on Circulation of Medical Devices	166; 2017	Chapter 4, Article 16 states that trials of medical devices are carried out in accordance with national laws. However, it is not clear what type of research on/with medical devices is meant in this document.
The Criminal Code of the Kyrgyz Republic	127; 2021	Article 147 states that conducting trials without the written consent of an individual (or their legal representative) or otherwise illegally conducting clinical trials of medicines, which negligently entailed significant harm, shall be punishable by a fine of 200 to 500 minimum monthly wages (or 500–1000 wages if grave harm), with deprivation of the right to hold certain positions or engage in certain activities for a term up to three years (for five to eight years if act committed against child or disabled person), or by community service from forty to one hundred hours.

According to the law, a preclinical (non-clinical) study is defined as a chemical, physical, biological, microbiological, pharmacological, toxicological, or other types of experimental study or a series of studies on a substance (medicine) by applying scientific assessment methods to study a specific action and/or evidence of safety for human health. A clinical study (trial) of a medicinal product is described as any study involving a human participant, aimed at identifying or confirming the clinical, pharmacological, and/or pharmacodynamic effects of one or more investigational medicinal products and/or identifying adverse reactions to one or more investigational medicinal products, and/or to study the absorption, distribution, metabolism, and excretion of one or more drugs to confirm its (their) safety and/or effectiveness.

Written consent to participate in clinical trials is mandatory according to the law and must mention relevant information including the nature, objectives, potential benefits and risks of the clinical trials and the medicinal product, the procedure for conducting clinical trials, and rights and obligations of a person participating in clinical trials. Furthermore, it is mandatory to ensure the life and health of a person participating in clinical trials. Insurance should be guaranteed and funded by the party conducting the clinical trial or the financing party per civil law. The law further requires the maintenance of a list of the institutions, organizations, and enterprises, including medical institutions and organizations that have been authorized to conduct research trials of medical devices/products for their registration.

### Regulations and Model Contracts

Regulations are the implemented rules created by the government's executive body to ensure that legislation is enforced in practice. Most often they are tied to the law but incorporate much more detail. A model contract is a template for agreements between the government and companies. Some governments have formalized the use of the model contract by establishing it as a rule or by annexing it to legislation. In other countries, the model is more of a guiding document created by the government or state-owned enterprise(s) responsible for licensing or contracting (NRGI, 2015).

Kyrgyzstan signed the accession agreement to join the Eurasian Economic Union (EAEU) in 2014, followed by its accession in 2015. The country is part of the EAEU's single market for medicines and medical devices and follows the common regulations. The regulations state that the main task of the REC is to protect the rights and health of the participants, as well as to guarantee their safety in clinical trials, especially in cases where participants gave consent due to their heightened expectations of the perceived benefits of participation (Table 2).

**Table 2.** Regulations That Govern Scientific Research Ethics in Kyrgyzstan.

Regulation	Regulation Number; Year of Adoption	Description
Technical regulation on the safety of medicines for medical use	137; 2011	Defines the mandatory requirements for medicinal products and regulates relations arising in the process of development, production, manufacture, storage, transportation, sale, and disposal of medicinal products. Apart from preclinical and clinical studies, the document describes non-interventional studies in which drugs are prescribed in the usual way in accordance with the conditions specified in the registration dossier. It further describes the pillars of informed consent and research ethics. Annex 2 describes the Regulations on Good Laboratory Practice (GLP) for conducting preclinical (non-clinical) studies on the safety of biologically active substances and drugs in laboratory animals or “in vitro”. Annex 3 describes the Regulations on Good Clinical Practice (GCP) for clinical trials.
The decree of the Government of the Kyrgyz Republic “On some issues related to state registration in the field of circulation of medicines”	405; 2018	Common to the Commonwealth of Independent States (CIS) member states, new regulations standardize the main stages of the product circulation cycle. The new EAEU rules include (On Approval of the Rules of Good Clinical Practice 2016): Rules of Good Clinical Practice of the Eurasian Economic Union, approved by the decision of the Council of the Eurasian Economic Commission dated November 3, 2016, No. 79 Rules of Good Laboratory Practice of the Eurasian Economic Union in the field of drug circulation were approved by the decision of the Council of the Eurasian Economic Commission dated November 3, 2016, No. 81 Rules for Conducting clinical and clinical laboratory tests (research) of medical devices approved by the decision of the Council of the Eurasian Economic Commission dated February 12, 2016, N 29.

### Contracts

Contracts are agreements between two or more parties. If the laws are in effect throughout the territory, a contract applies only to a certain place and to the entities that are parties to it. They set out those roles and responsibilities that are not specified in the law and often include terms and conditions relating to operational, financial, social, environmental, and performance obligations (NRGI, 2015). Examples include the organizational-legal work charter governing RECs. Accordingly, we identified eight RECs operating in Kyrgyzstan. As shown in Table 3, only two RECs were found to be registered with the Office for Human Research Protections (OHRP, USA). Furthermore, only one REC was found to be registered with the Food and Drug Administration (FDA, USA).

**Table 3.** Eight RECs That Provide Approval for the Scientific Research in Kyrgyz Republic.

REC Name	Description
Committee on Bioethics of Ministry of Health, Kyrgyzstan	Conducts reviews of preclinical and clinical trials of medicines, medical products, and medical devices.
Committee on Bioethics of I.K. Akhunbaev Kyrgyz State Medical Academy	Conducts reviews of biomedical research involving humans and animals.
Ethics Committee of the National Institute of Public Health (*formerly Scientific-industrial union “Preventive Medicine” of the Ministry of Health KR*)
Bioethics Committee of the International School of Medicine at the International University of Kyrgyzstan	–
Ethics Committee of the M. Mirrakhimov National Cardiology and Therapy Center	Reviews clinical studies of new technologies and drugs.
Committee on Biomedical ethics of the Osh State University of the Kyrgyz Republic	–
Committee on Bioethics of the Global Research Institute (GLORI Foundation) in the Kyrgyz Republic	Conducts reviews of behavioral studies or sociological surveys. OHRP registered. Refers to Belmont Report as guiding document for review process.
Institutional Review Board of the American University in Central Asia in the Kyrgyz Republic	Refers to Belmont Report as guiding document for review process.

## Discussion

Our results show that legislation in Kyrgyzstan does not provide adequate guidance to the RECs on their role and conduct during public health emergencies. We identified major gaps in the country's ethical research regulations. Clinical trials of medicines and medical devices are the only types of research that are legally subject to ethics review in the country. A wide range of other research, both biomedical and non-biomedical, involving human participants is currently unregulated by national legislation. This problem is indicative of the attitude of non-equivalence and neglect that has been adopted between the rather stringent ethical review requirements for clinical trials and the relatively weak requirements for other types of research.

Research involving humans that does not fall within the purview of biomedical research, including sociological, anthropological, psychosocial, and community-based (“non-biomedical”) research, is the least regulated at the national, regional, and international levels. In most cases, there are no mandatory ethical standards governing such research studies, irrespective of the level of risk they might pose. Such practices are especially prone to exploitation and misuse in public health (Gefenas et al., 2010). Public health research is often seen as falling outside the basic bioethical principles that govern biomedical research and clinical trials designed to protect human participants (Bayer & Fairchild, 2004; Lee, 2017). The specificity of public health research calls for a separate set of ethical rules that are formulated in accordance with local conditions and international regulations.

For instance, scientific studies involving the collection and analysis of identifiable health data by public health authorities generates knowledge that doesn’t directly benefit the participating population but carries the risks of participation (James & Lawrence, 2004). In addition, due to their networked nature (especially in the case of studies that involve the use of social media platforms), these methods are fraught with ethical issues and suffer from a lack of clear consensus on ethical handling of such data (Hunter et al., 2018). Consider studies using mass screening to report the spread of COVID-19. Case reporting forms (CRFs) have been used to standardize and share data with international authorities, including the WHO. Whilst such forms cannot directly identify a patient, they do request sensitive patient data, including demographic (gender, age) and medical (co-morbidity, diagnosis) information. This leads to an overriding obligation to breach confidentiality (Al Tajir, 2018) when sharing data as CRFs are mandatory for member countries. In addition, researchers are not able to anticipate in advance whether the data they have collected will be used for secondary studies.

This is pertinent because, in a public health emergency, the informed consent of participants is often vague and incomplete, thereby raising the issue of individual versus public rights (Ballantyne, 2019). Individual informed consent is particularly difficult to obtain for such studies (e.g., patients in red zones, undergoing economic/social stress, or in a confused unclear state of mind). Furthermore, concerns have been raised regarding the need for ways to regulate qualitative research involving telephone/video-supported interviews and to maintain privacy, confidentiality, and data security. Ensuring the safety of researchers and participants of a study in red zones, and protection from media speculation/infodemics are other pressing issues that need to be considered by the RECs. Kyrgyzstan, for example, recruited medical students during COVID-19 to relieve the health system (Dzushupov et al., 2021), a strategy burdened with its own ethical dilemmas.

Ethical issues remain regarding how to use and store biological tissues and samples. In mass disasters, various destructive agents may cause tissue changes. Identification of such changes is essential in determining the cause of death, diagnosis of illness, and accurate data registration. Tissues also help to understand disease processes and improve medical care. According to Article 43 of the “Law on the protection of the health of citizens in the Kyrgyz Republic”, an autopsy cannot be performed if the deceased's family or legal representative or the deceased's will prohibits it, except in cases of suspected violent death. In addition, the list of diseases for which an autopsy is mandatory is determined by the Ministry of Health (Decree no. 492; 2005). However, in practice, the law on relatives’ consent overrides mandatory autopsy (National Committee on Confidential Enquiry into Maternal Deaths, 2014). Religious beliefs and the desire to reduce the number of recorded misdiagnoses have limited the number of autopsies performed in the country.

According to the European Union's IncoNet CA report, Kyrgyzstan's research infrastructure has stagnated for decades, suffering from outdated equipment, lack of funding, and an aging scientific workforce (Spiesberger et al., 2016), leading to the closure of several histology centers. This neglected attitude is further exemplified by the failure to adopt various international best practice guidelines governing non-biomedical research, a problem common to most other Commonwealth of Independent States (CIS) Member States (Table 4). The legal requirements and the function of the RECs need to be better communicated in terms of the review of non-biomedical studies with a commitment to greater community engagement and partnerships, as well as the protection of human participants. Even amongst the already implemented laws, there seem to be contradictions that require further clarifications. Previous research has highlighted how to combine this commitment to community involvement with human subject protection in public health research in developing nations (Emanuel et al., 2004).

**Table 4.** Number of OHRP and FDA Registered Research Ethics Committees Amongst CIS Members (as of Jan 2023).

CIS Member Country	OHRP only registered	FDA only registered	OHRP & FDA registered	Total Active	Deactivated
Armenia	3	–	1	4	12
Azerbaijan	1	–	–	1	1
Belarus	3	–	1	4	26
Kazakhstan	3	–	2	5	18
Kyrgyzstan	1	–	1	2	4
Moldova	5	–	2	7	8
Russian Federation	36	1	8	45	175
Tajikistan	2	–	–	2	4
Turkmenistan	–	–	–	–	–
Ukraine*	16	1	14	31	123
Uzbekistan	1	–	–	1	1

*Ukraine has one more research ethics committee which is under “initializing” phase of registration.

Further examination of the available documents reveals major regulatory inconsistencies. While the “Law on the Circulation of Medicines” and “Law on the Circulation of Medical Devices” forbid clinical trials on minors, pregnant women, and people with mental disorders, the “Technical Regulation on the Safety of Medicines for Medical Use” makes exceptions for the participation of these vulnerable groups. In its Annex 3, the technical regulation states that studies on children may be authorized if the use of the product is exclusively for the treatment of children's diseases or to obtain data on the optimal dose of the product for the treatment of children (in the latter case, trials in minors must be preceded by studies in adults). There is no explicit requirement for “assent” from minors, although written parental/legal guardian consent is required. Similarly, pregnant women may be enrolled in trials if the necessary information can only be obtained from clinical trials of drugs on pregnant women and when the risk of harm to a pregnant woman and fetus is completely excluded. Such conflicting interpretations have created confusion for RECs and made it difficult to provide the necessary approvals, even though there are exceptions in the legislation for clinical trials in the fields of pediatrics, obstetrics, gynecology, and psychiatry.

Another conflict arises when allocating scarce resources. Take, for example, ventilators allocated during the COVID-19 pandemic. The national health infrastructure was severely strained, with 50% of the ventilators inoperable and 25% of the recorded cases in health workers (Dzushupov et al., 2021). In these circumstances, resource allocation is usually guided by some form of prioritization (either based on the severity of illness, patient status, or the individual's perceived role in society), in which the health benefits to the population take precedence over the ethical duties of the clinician towards the rights of the individual patient (Tian, 2021). Such guidelines directly contradict Kyrgyz laws, which state that individual trial participants’ interests must override scientific and societal interests. Using age to determine priority for ventilation allotment, some studies have reported that the public prefers younger patients (< 40 years) as a priority (Huseynov et al., 2020). Such preferences are justified by the ethical principles of maximization of social and individual benefits and prioritization of the worst-off. For decision support, decision-making power is often outsourced to triage committees (or hospital RECs) that have limited clinical expertise and first-hand information about patients. This has the potential to introduce bias and violate the principle of equity (Tian, 2021). In such situations, the adoption of these criteria may systematically exclude (or bias) certain populations.

Despite the clear lack of regulatory guidelines in Kyrgyzstan on how RECs should conduct research in public health emergency situations, all the guidelines developed and adopted by various international bodies (like WHO and PAHO) are available to RECs in Kyrgyzstan. Therefore, Kyrgyzstan has no ethical regulatory vacuum in this area. The problem is that when ethical principles and guidelines are not adopted and followed, there are limited opportunities to act against the perpetrators. More importantly, there is no common platform for the education of Kyrgyz researchers on ethical issues in public health emergency research. It seems that the system is stuck in a vicious cycle where RECs demand showcasing the wider need for adoption of such guidelines whilst the researchers resist applying due to obsolete equipment, lack of funding, patient resistance, and bureaucratic paperwork. The net effect is the degradation of the existing scientific ecosystem in the country.

Herein, we aggregated key points from various guidelines and resources that the RECs can refer to when assessing proposals concerning public health emergencies in conjunction with national guidelines (Mezinska et al., 2016; SAMHSA, 2016; WHO, 2020a, 2020b, 2022):

- **Centralization of the review process** – RECs are encouraged to coordinate with other national and international RECs to identify any potential overlap in research proposals. A central or dedicated REC can be formed at national or regional level for improving efficiency and maximizing knowledge (Collogan et al., 2004).
- **Alternative review mechanisms** – A prioritization scheme should be adopted to assess and determine which proposals could be eligible for expedited processing and don’t require a full review. Alternative strategies such as approving just-in-case generic protocols with a skeletal outline of different study designs can also be considered. Such templates can be used by researchers during emergencies and for funding applications. Prioritization of protocols by specific expert RECs, rolling review of protocols (in part or full), and individual review by the chair of the REC are other suggested mechanisms (Tansey et al., 2010).
- **Synthesize evidence, prevent duplications, and prioritize new research** – Literature reviews and evidence gathering by researchers should be encouraged. RECs should ensure that research proposals are supported by sufficient evidence and that research gaps are identified while avoiding potential duplications. Several databases can be consulted, including PROSPERO, the Cochrane Library, the Living Overview of Evidence (L.OVE), and indexing services such as PubMed, Scopus, and Web of Science.
- **Analyze risks and benefits to the participants** – RECs should ensure that the included participants are safeguarded whilst considering the novelty of the research question and its necessity to the field. Involvement of participants in multiple studies should be evaluated carefully and be guided by utilitarianism and social justice. Research is needed for refining future disaster responses and promoting systemic inequities including improper resource allocation (Ferreira et al., 2015). The MEURI (Monitored Emergency Use of Unregistered and Investigational Interventions) framework could provide a base set of regulations for RECs when considering proposals investigating unregistered and experimental interventions during public health emergencies.
- **Informed Consent** – RECs should make sure that protocols require collection of informed consent. Furthermore, verifying the decisional capacity of the participants is essential before allowing participation. Multiple scales are available for use including the University of California, San Diego Brief Assessment of Capacity to Consent (UBACC) and the MacArthur Competence Assessment Tool for Clinical Research (McCAT-CR). Additionally, the behavioral indicators should be checked. RECs should review that protocols are not coercing participation by providing monetary benefits or biasing participants towards a moral sense of civic responsibility after a disaster (Collogan et al., 2004). Finally, collection, storage, future use, bio-banking and export of human biological material or data should be explicitly stated and explained.
- **Consider cultural awareness and sensitivity** – RECs should ensure that protocols do not add to the distress of the participants and utilize methodologies like interviews that are seen more welcoming (Legerski & Bunnell, 2010). The researchers should demonstrate that cultural beliefs and sensitivities were considered and accounted for whilst designing the research protocol. Translations of international protocols should be made by language experts. Access to mental health specialists for consultation could be considered as well. Experimental protocols should be considered with extreme precaution and only validated protocols should be encouraged.
- **Researcher Training** – RECs should ensure that the researchers undertake an ethics module on conducting research in emergencies (Allden et al., 2009). It is important that the researchers are aware of safe locations in case of earthquakes, floods, or other public health emergencies. The public should be educated and made aware about the proper usage of personal protective gear. RECs should ensure measures have been taken by researchers to avoid overgeneralization of the results. Research should not attract undue attention or publicity.
- **Participation with relief organizations** – Collaboration with relief organizations like the Red Cross, Doctors without Borders, WHO, PAHO, or National Disaster Relief Forces can ensure that the safety of both participants and researchers is accounted for. Furthermore, such collaborations would ensure that scientific research does not hinder relief operations.
- **Ethical participant selection and resource allocation** – RECs should review that the selection of the participants and resource allocation is based on scientific reasons and not priority or worse-off basis. Exploitation of participants should be prevented, and protocols should ensure that the end results of the study would benefit the included participants (Jennings & Arras, 2008). Regarding resource allocation, RECs could encourage adoption of the “Reserve Model” whereby patients can be grouped into different sets (e.g., young or old) and for each group, a fixed number of resources is reserved whilst the remaining unreserved resources are open for all community members. When assigning those fixed resources, a score-based scale might be used (Pathak et al., 2020).

In conclusion, new principles or rules are urgently needed to engage communities in a constructive partnership during the organization and implementation of public health research (Childress et al., 2002). The solution, however, does not lie in simply changing or reinterpreting the rules. Rather, it requires a new way of thinking about the relational nature of scientific research (Quinn, 2004).

## Best Practices

RECs must have a legal framework for reviewing public health research that considers the unique challenges and concerns associated with protecting and respecting community autonomy. There is a need to create national legal frameworks aimed at reducing ethics dumping and exploitation of participants and their data. A new ethics regulation program that is more appropriate for public health research would increase the preparedness and effectiveness of RECs during such situations.

## Research Agenda

The recent increase in public health emergencies has highlighted an urgent need for being better prepared to address relevant research questions in the context of public health emergencies like pandemics, epidemics, and natural disasters. Accordingly, RECs must be capable of operating in such emergencies. In our study, we explored the legislative measures governing RECs in Kyrgyzstan.

## Educational Implications

Existing national ethics review procedures and ethics committee guidelines are overwhelmingly biomedical in nature, rarely considering the shared experiences of community research. They primarily focus on the principle of risk assessment for individuals rather than communities. Furthermore, it is not only the capacity of RECs to provide an adequate review that seems to be missing but also the systematic exclusion and operational undermining of community-based research that could endanger the participants. This highlights the neglect of non-biomedical research in the country, rather than necessarily being a contrast between community-based research and individual human participant protection.
